# An 8-step approach for the systematic development of an evidence-based exercise program for patients undergoing hematopoietic stem cell transplantation

**DOI:** 10.3389/fonc.2023.1132776

**Published:** 2023-04-18

**Authors:** Ki-Yong An, Mi-Seong Yu, Wonhee Cho, Meeok Choi, Kerry S. Courneya, June-Won Cheong, Justin Y. Jeon

**Affiliations:** ^1^ Faculty of Kinesiology, Sport, and Recreation, University of Alberta, Edmonton, AB, Canada; ^2^ Department of Sports Industry Studies, Yonsei University, Seoul, Republic of Korea; ^3^ Department of Exercise Science, Syracuse University, Syracuse, NY, United States; ^4^ Department of Nursing, Yonsei University College of Medicine, Seoul, Republic of Korea; ^5^ Department of Internal Medicine, Yonsei University College of Medicine, Seoul, Republic of Korea; ^6^ Cancer Prevention Center, Yonsei Cancer Center, Shinchon Severance Hospital, Seoul, Republic of Korea; ^7^ Exercise Medicine Center for Diabetes and Cancer Patients (ICONS), Seoul, Republic of Korea

**Keywords:** exercise, cancer, hematopoietic stem cell transplantation, evidence-based design, fitness

## Abstract

**Background:**

A tailored and reliable intervention program developed based on evidence is necessary for patients with serious health conditions.

**Objective:**

We describe the development of an exercise program for HSCT patients based on evidence from a systematic process.

**Methods:**

We developed the exercise program for HSCT patients using eight systematic steps: (1) a literature review, (2) understanding patient characteristics, (3) first expert group discussion, (4) development of the first draft of the exercise program, (5) a pre-test, (6) second expert group discussion, (7) a pilot randomized controlled trial (n=21), and (8) a focus group interview.

**Results:**

The developed exercise program was unsupervised and consisted of different exercises and intensities according to the patients’ hospital room and health condition. Participants were provided with instructions for the exercise program, exercise videos *via* smartphone, and prior education sessions. In the pilot trial, the adherence to the exercise program was only 44.7%, however, some changes in physical functioning and body composition favored the exercise group despite the small sample size.

**Conclusion:**

Strategies to improve adherence to this exercise program and larger sample sizes are needed to adequately test if the developed exercise program may help patients improve physical and hematologic recovery after HSCT. This study may help researchers develop a safe and effective evidence-based exercise program for their intervention studies. Moreover, the developed program may benefit the physical and hematological recovery in patients undergoing HSCT in larger trials, if exercise adherence is improved.

**Clinical trial registration:**

https://cris.nih.go.kr/cris/search/detailSearch.do?seq=24233&search_page=L, identifier KCT 0008269.

## Introduction

Although hematopoietic stem cell transplantation (HSCT) is a primary medical treatment option for patients with hematologic diseases such as leukemia, lymphoma, and multiple myeloma (1), it has many physiological and psychological side effects (1–4). Previous randomized controlled trials have reported that exercise improves physical function (5–7), fatigue (7, 8), depression (9, 10), anxiety (9), and quality of life (11) in patients receiving HSCT. However, the exercise programs described in previous studies have limited applicability to HSCT patients in real clinical settings in terms of different treatment processes and environments (e.g., isolation period, size of the hospital room, and access to exercise equipment and therapist). For example, aerobic exercises on a treadmill or stationary bike that were used in previous studies (5–7, 11–18) require space and equipment. Moreover, the supervised exercise programs (7, 15–18) are difficult to implement in an isolated room due to limited access.

Intervention programs must be cautiously and carefully developed in clinical trials. Particularly for patients receiving intense treatment or with serious health conditions such as HSCT, a reliable intervention program which guarantees safety and feasibility is required. Evidence-based medicine involves integrating the clinical expertise, proficiency, and judgment that individual clinicians acquired from clinical experience and practice, with the best available external clinical evidence from systematic research (19–21). Therefore, the purpose of this study was to develop an evidence-based inpatient exercise program for patients undergoing HSCT using a systematic process used by An et al. (21). Here, we describe the development process of an evidence-based exercise program for patients receiving HSCT.

## Methods

### Study design and participants

In the current study, we developed an exercise program using 10 systematic steps developed by An et al. (21). We changed the order of the focus group interview from the original process and included only the first eight steps. The development process in this study included (1) a literature review, (2) understanding patient characteristics, (3) first expert group discussion, (4) development of the first draft of the exercise program, (5) a pre-test, (6) second expert group discussion, (7) a pilot study, and (8) a focus group interview. Patients with hematologic disease receiving HSCT participated in this study. The purpose of the eight-step procedure below was to develop a safe, reliable, and effective exercise program based on solid evidence.

### Step 1. Literature review

We conducted a literature review as the first step. The purpose of this step was to collect evidence on exercise programs for HSCT patients from previous studies. The electronic online literature databases such as Embase, Pubmed, and CENTRAL were searched. To identify relevant studies, all available articles that were published before March in 2016 were searched using the following search terms: (hematopoietic stem cell transplantation OR bone marrow transplantation OR autologous transplantation OR allogeneic transplantation OR peripheral blood stem cell transplantation) AND (exercise OR physical activity (PA) OR training) AND (randomized controlled trial). Two investigators (KYA, MSY) independently filtered the references retrieved from the literature search by screening the titles and abstracts. The selected articles were re-filtered based on full-text review to identify relevant information. The articles were included if they met the following inclusion criteria: 1) published in English, 2) full-text article, 3) randomized controlled trial, 4) included inpatient exercise intervention, and 5) included patients with hematologic diseases receiving HSCT. The literature search adhered to the Preferred Reporting Items for Systematic reviews and Meta-Analyses statement guidelines.

### Step 2. Understanding patient characteristics

The purpose of step 2 was to collect evidence on patients’ performance status, physical activity levels, and opinions about exercise directly from the patients. In this step, we collected data by observation, survey, interview, and physical assessment during patients’ hospitalization. An exercise therapist accompanied a hematologist on the daily ward rounds, and observed and recorded the changes in patient performance status from the treatment process by the Karnofsky Performance Status Scale (KPS) (22) for four weeks.

The patient survey was conducted to identify the PA level, performance status, exercise barriers, and exercise beliefs of HSCT patients. Pre-diagnosis and current PA level were assessed by the International PA Questionnaire short form (23), while exercise belief was assessed using the modified Exercise & Quality of Life Questionnaire (24). Exercise barriers were investigated using an open question and performance status was assessed by the KPS (22).

Participants were also interviewed about their exercise barriers and preferences, expected effects of exercise, and changes in physical and psychological condition according to treatment during the hospitalization. A face-to-face interview was conducted with trained research staff in an independent space to ensure participant comfort.

Range of motion at eight joints (i.e. neck, shoulder, elbow, wrist, trunk, hip, knee, and ankle) was assessed using goniometers and patient-perceived pain levels during each movement were recorded using the Wong-Baker Faces Pain Rating Scale (0–10) (25).

### Step 3. First expert group discussion

The purpose of this meeting was to collect experts’ opinions about patients’ performance status and health conditions which are important information to develop a draft of the exercise program. An expert group consisted of two hematologists, a nurse, and a fellow in hematology, and three exercise specialists including one professor in kinesiology. Patients’ condition and physical functions, exercise goals, and precautions for exercise intervention were discussed.

### Step 4. Development of the first draft of the exercise program

A draft version of the exercise program was developed based on the results of steps 1 to 3. First, possible resistance and stretching exercises for main muscle groups according to the anatomical motions of each joint were identified. Thereafter, the necessary and feasible exercises to meet the exercise goals were selected. Finally, the most appropriate exercises for HSCT patients varying intensities, ranging from extremely low to moderate, were included in the draft considering the patients’ conditions, exercise precautions, and space availability.

### Step 5. Pre-test

The purpose of the pre-test step was to examine the feasibility and safety of the draft program with three HSCT patients before we completed the final program. Participants learned tailored exercises and were provided with an exercise brochure, exercise videos, and daily log book before they entered the isolated room (i.e. bio-clean room or laminar air flow room) for HSCT. The participants were asked to perform the exercise program for approximately three weeks while staying in the isolated room. The adherence to the program was recorded in the log book.

### Step 6. Second expert group discussion

In this meeting, the expert group discussed any changes and additions to the draft program. Specifically, they discussed the intensity, feasibility, safety, suitability, and effects of each exercise based on the results of the pre-test.

### Step 7. Pilot randomized controlled trial

The purpose of the pilot RCT was to assess feasibility, acceptability, adherence and safety in the small number of patients undergoing HSCT. Examining the effects of the developed exercise program on health-related fitness and hematological recovery is exploratory. For recruitment, a nurse who worked with physicians sent the eligible patients list to the research coordinator, then the research coordinator visited the patients to explain the study and to ask to participate in the study with a written informed consent form. A total of 21 participants with hematologic diseases who were initiating HSCT in the Severance hospital in Seoul, South Korea were recruited and randomly assigned to either the exercise group or the usual care group in a 1:1 ratio by the research coordinator using the permuted block design considering sex and transplantation type. Participants were randomized after baseline assessment but for the post-intervention test, testers were not blinded to group allocation. Participants in the exercise group learned the exercise program prior to admission to the isolated room and were asked to perform the exercise program during their hospitalization. Printed information and video on exercise (https://youtu.be/2TyjelwsrvU) along with a daily log book were provided. Exercise specialists (KYA, MSY) visited the participants 2-3 times per week and encouraged participants to comply with the prescribed exercise. The exercise specialists were certified for exercise training and were in master’s and doctoral programs in Exercise Medicine, respectively. Body composition, physical function, and symptoms were assessed before and after their stay in the isolated room. Blood transfusion volume and time to engraftment were obtained from each patient’s electronic medical record. The paired t-test was used to compare baseline and post-intervention assessments in each group. The independent t-test was used to compare differences between groups.

### Step 8. Focus group interview

We also collected qualitative data on the barriers and facilitators to completion of the exercise intervention during HSCT. A total of six participants in the exercise group of the pilot RCT participated in a face-to-face interview. All interviews were recorded and then transcribed and summarized by an interviewer, and the transcript and summary of the interview were confirmed by each participant to ensure their accuracy. The method of the qualitative data collection has been reported elsewhere (26).

## Results

### Step 1. Literature review

**Figure 1:**
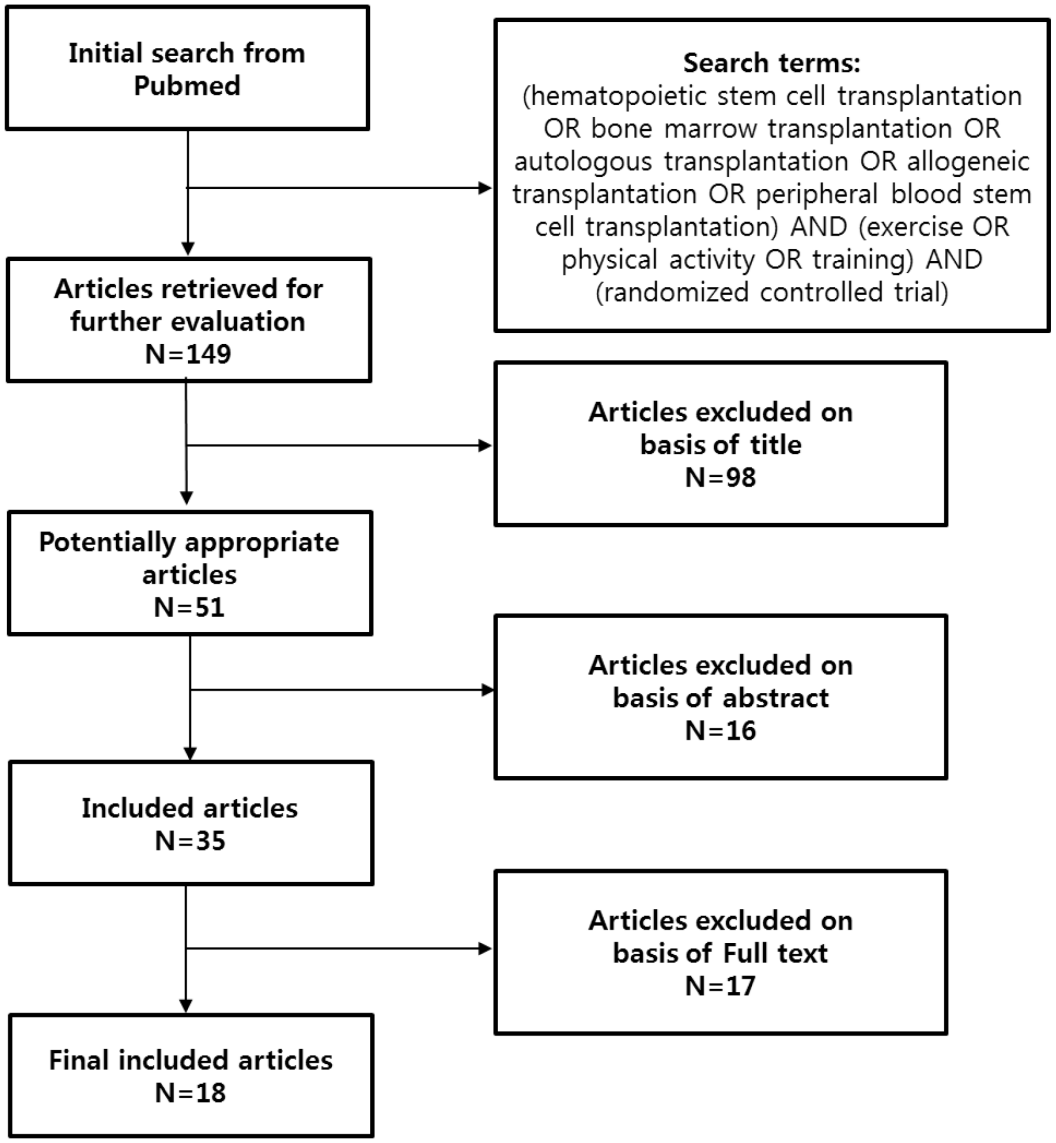
The literature search protocol.

A total of 149 articles were initially found in the literature search and 18 articles (11 intervention programs) were included in the literature review. The literature search protocol is described in **Figure 1**, and the characteristics of the exercise programs in the included articles are shown in **Supplementary Table 1**.

The literature review identified that the previous exercise programs included aerobic, resistance, breathing, core, and stretching exercises, and an elastic band, bare hand, or ankle weight were used for the resistance exercise and a stationary bike or treadmill walking was used for the aerobic exercise. Stretching was performed as a warm-up or cool-down exercise in most of the included studies.

Exercise intensity was 50–70% of the heart rate (HR) reserve or rate of perceived exertion (RPE) of 10–14. The intensity of the resistance exercise was 40–60% of 1 repetition maximum, an RPE of 10–16, or slightly strenuous to strenuous. Exercise duration was gradually increased in the range of 20–40 min/day. The range of exercise frequency was 3–7 days/week. Among 11 programs, seven were supervised interventions. We identified the possible type, intensity, and frequency of exercise that HSCT patients can perform.

### Step 2. Understanding patient characteristics

A total of three HSCT patients were observed for four weeks. Their KPS scores were in the range of 20–70 depending on the treatment process and individual health condition. The patients who had KPS scores of 20–30 spent most of their time lying on the bed in the laminar air flow/bio-clean room. Participants initially stayed in the general room for about a week, followed by a stay in the laminar air flow or bio-clean room of about three weeks for chemotherapy, transplantation, and engraftment. After engraftment, they moved back to the general room and stayed there for one or two weeks before discharge. The room type (laminar air flow or bio-clean) is generally decided by transplantation type (autologous or allogeneic). Participants in the laminar air flow room were restricted from contacting with others and leaving the room to prevent infection. The patients in the bio-clean room have a caregiver in the room but the bed space is sterile and covered with plastic curtains to minimize contact with other people. The patients in the bio-clean room had to stay on the bed within the plastic curtain whenever possible except when going to the bathroom. Thus, the PA levels of the patients were extremely low. The results of the observation were summarized as follows: 1) exercise intensity should be started very low; 2) exercises that can be performed on the bed are required because of the limited ambulation; 3) an unsupervised exercise program was needed because of the limited contact; and 4) recovery of physical function, maintenance of muscle mass and range of motion, and improvement of psychological factors might be the primary goals of the exercise program.

A total of 15 HSCT patients were surveyed. The results of the patient survey are shown in **Table 1**. The mean current light PA time was shorter than the pre-diagnosis light PA. No participants had current moderate to vigorous PA and participants with multiple myeloma were the least active. The KPS score during chemotherapy was lower than those in the pre-chemotherapy and the recovery period. Specifically, in pre-chemotherapy period, three participants (75%) reported the KPS score 60 (i.e., requires occasional assistance, but it able to care for most of his personal needs) and one participant (25%) reported the score 90 (i.e., able to carry on normal activity; minor signs or symptoms of disease). The one participant (100%) during chemotherapy reported the score 50 (i.e., requires considerable assistance and frequent medical care). In the recovery period, the performance status varied (40 to 80) and it improved over time. Four participants (40%) reported the score 60, two participants (20%) reported the score 70 (i.e., cares for self; unable to carry on normal activity or to do active work), and two participants (20%) reported the score 40 (i.e., disable; requires special care and assistance). Dry mouth, fatigue, drowsiness, numbness, and pain were the most common symptoms. All participants reported that they believed that exercise positively affects the recovery from HSCT.

**Table 1:** Participants’ physical activity level and performance status.

	Pre-diagnosis daily walking time (min/day)	Current daily walking time (min/day)	Karnofsky score (0~100)
Total (n=15)	27.86 ± 38.47	1.74 ± 4.12	60.67 ± 13.35
Diagnosis			
Lymphoma (n=7)	40.36 ± 51.87	3.02 ± 5.69	62.86 ± 16.04
Leukemia (n=5)	23.65 ± 20.88	1.00 ± 2.24	62.00 ± 10.95
Multiple myeloma (n=3)	5.71 ± 9.90	0	53.33 ± 11.55
Stem Cell Transplantation			
Autologous (n=8)	4.29 ± 8.57	0	50.00 ± 11.55
Allogenic (n=3)	22.72 ± 19.89	6.67 ± 7.64	70.00 ± 10.00
Haploidentical (n=4)	32.62 ± 31.48	0	60.00 ± 10.00
Treatment Process			
Pre-chemo therapy (n=4)	22.32 ± 21.65	1.54 ± 2.79	67.50 ± 15.00
Chemo therapy (n=1)	145.50 ± 0.00	0	50.00 ± 0.00
^*^Recovery (n=10)	18.32 ± 22.24	2.00 ± 4.83	59.00 ± 12.87

Values are mean ± SD.

^*^Recovery means the patient’s status after being discharged from a bioclean/laminar flow room.

A total of 14 participants completed the interview. We identified that participants experienced physical symptoms such as nausea, vomiting, exhaustion, and anemia during chemotherapy and recovery period as well as psychological distress due to loneliness and claustrophobia in the isolated room and wanted a daily exercise program because of boredom and loneliness. Additionally, the participants thought that resistance exercise, stretching, and stepping were appropriate during HSCT treatment and expected those exercises would help them maintain muscle mass, muscle strength, and physical fitness as well as improve their blood circulation. The exercise barriers included limited space, lack of information, decreased physical fitness, and the presence of medical tubes and intravenous lines. In particular, the patients with multiple myeloma reported bone-related problems such as dislocation and severe pain that were caused by osteolytic lesions at the shoulder or lower back. Participants additionally wanted an exercise program that featured: 1) reliable exercise information; 2) supervised exercise with an expert; 3) therapeutic exercise programs as a standard treatment; 4) tailored and modified exercise intensity according to individual daily condition; 5) exercises that could be performed in a limited space; 6) sufficient pre-exercise education before entering the isolated room; and 7) availability of self-exercise materials in the isolated room.

A total of nine participants participated in in the measurement of the range of motion. Two participants with multiple myeloma were unable to complete the measurement because of osteolytic lesion–related problems. The other seven participants had full range of motion without any limitations or pain at all joints. This test identified that patients with multiple myeloma commonly had osteolytic lesions that limited their movement, while other HSCT patients did not have any limited movements for exercise.

### Step 3. First expert group discussion

The hematologist (JWC) suggested different types of exercise for the three different hospital rooms because of different spaces and contact limitations, and for participants with multiple myeloma because of osteolytic lesions. The kinesiology professor (JYJ) suggested different intensities of the exercise program according to an individual’s daily condition, and extra exercises to strengthen the joints of participants with multiple myeloma. He also suggested a daily exercise because the exercise intensity would be low. The exercise goal was deemed to be the maintenance of muscle mass, muscular fitness, walking ability, and cardiopulmonary function, all of which may decline due to lack of PA and immobilization in the isolated room. Aerobic exercise such as walking or biking was not included in the program due to the limited equipment and space. Instead, rest intervals between exercises were minimized to increase heart rate during exercise.

### Step 4. Development of the first draft of the exercise program

The exercises included stretching, resistance exercises, and joint-specific exercises using body weight or an elastic band. The exercises were performed in three different positions (supine, sitting, and standing) according to patient condition (bad, normal, or good) and hospital room type (general, laminar air flow, or bio-clean room). Three extra joint exercises (shoulder, lower back, and knee) were included for patients with osteolytic lesions.

### Step 5. Pre-test

A total of three participants participated in the pre-test: two in the bio-clean room and one in the laminar air flow room. Participants had no problem performing exercises following the instruction of the brochure and exercise videos. Participants reported that all exercises in the program were feasible and safe when their physical condition was good. However, they reported difficulty completing the prescribed exercises daily when their physical condition was poor, even though exercise prescribed to them was extremely low- or low-intensity exercise program which they could perform in the supine position. A lack of motivation and energy to comply with the prescribed exercise program were the most significant barriers to exercise. The participants reported that they laid on bed all day when their physical condition was extremely bad.

### Step 6. Second expert group discussion

In the second expert group discussion, all expert group members agreed to use the first draft of the exercise program without changes for the next step.

### Step 7. Pilot RCT

**Table 2** reports the baseline characteristics of participants for the pilot RCT. A total of 21 participants (exercise =10, control=11) were included in the trial. Males accounted for 50% and 45% and the average ages were 46.3 years and 53.7 years in the exercise group and control group, respectively. The results of pilot RCT are shown in **Table 3**. The average adherence to the exercise program was 44.7% which was calculated as the total number of completed sessions divided by the total number of planned sessions. The time for 8-foot up-and-go (*p*=0.032) and the symptom score (*p*=0.039) were significantly increased in the control group while there was no significant change in the exercise group. Moreover, there was a significant difference between groups for the time for 8-foot up-and-go (p=0.030). There were no significant differences between groups for other body compositions and physical functions but fat mass and fat percent tended to decrease more in the exercise group, and the 2-min knee up, hand-grip strength, and chair stand tended to decrease more in the control group compared to the exercise group. There was no exercise-related complication reported in the exercise group. These results showed the safety and potential positive effects of the exercise program on the patients’ health-related fitness, symptoms, and recovery-related variables. However, an advanced strategy to improve exercise adherence during HSCT is needed to optimize the effects of the exercise program.

**Table 2:** Participants’ characteristics for the pilot randomized controlled trial.

Variables	Total (n=21)	Exercise group (n=10)	Control group (n=11)	p-value
Sex (M/F)	10/11	5/5	5/6	0.84
Age (year)	50.2 ± 13.5	46.3 ± 12.8	53.7 ± 13.7	0.22
Diagnosis				0.50
Leukemia	4	3	1	
Lymphoma	4	1	3	
Multiple myeloma	11	6	5	
Myelodysplastic syndrome	1	0	1	
Amyloidosis	1	0	1	
Transplant				0.55
Autologous	16	7	9	
Allogenic^a^	5	3	2	

BMI, body mass index.

a Allogenic includes haploidentical transplant.

**Table 3:** The effects of the exercise program on recovery from HSCT.

Variables	n	Exercise group (n=10)			Control group (n=11)			*p* value	Δ
		Baseline	At discharge	Δ	n	Baseline	At discharge		
Body composition									
Weight(kg)	10	66.7 ± 17.0	64.4 ± 15.4*	-2.3 ± 2.6	10	58.8 ± 11.7	57.2 ± 11.9*	-1.6 ± 1.9	0.49
BMI(kg/m^2^)	10	24.1 ± 4.6	23.0 ± 3.9*	-1.1 ± 1.5	10	22.8 ± 3.6	22.1 ± 3.7*	-0.6 ± 0.8	0.37
Muscle mass(kg)	10	25.5 ± 8.3	25.1 ± 7.7	-0.5 ± 1.3	10	23.2 ± 4.9	22.3 ± 4.5	-0.9 ± 1.9	0.55
Fat mass(kg)	10	19.9 ± 5.6	18.2 ± 4.4	-1.7 ± 2.4	10	15.8 ± 4.2	15.8 ± 6.3	0.0 ± 2.8	0.16
Fat percent(%)	10	30.3 ± 6.3	28.8 ± 5.2	-1.6 ± 3.3	10	26.7 ± 3.5	26.8 ± 6.8	0.2 ± 4.7	0.36
BMR(kcal)	10	1379.3 ± 295.8	1367.6 ± 276.3	-11.7 ± 49.2	10	1298.4 ± 173.6	1264.4 ± 158.2	-34.0 ± 69.9	0.42
Physical function									
2-min knee up(reps)	7	96.0 ± 15.1	91.6 ± 22.0	-4.4 ± 12.8	8	94.0 ± 14.9	72.9 ± 35.7	-21.1 ± 31.8	0.22
Sit and reach (cm)	9	1.6 ± 11.1	-5.4 ± 10.5	-7.0 ± 10.7	11	1.7 ± 15.4	-1.8 ± 14.2	-3.5 ± 10.0	0.47
Hand-grip strength(kg)	9	27.6 ± 13.2	27.1 ± 11.0	-0.5 ± 3.5	11	26.0 ± 8.3	24.3 ± 7.4	-1.8 ± 4.4	0.48
Chair stand(reps)	9	13.4 ± 3.8	14.0 ± 1.7	0.6 ± 3.0	10	13.3 ± 2.4	11.7 ± 4.4	-1.6 ± 3.5	0.17
8 foot up and go(sec)	9	6.1 ± 1.2	5.9 ± 0.7	-0.1 ± 1.0	10	7.0 ± 1.6	8.7 ± 2.1*	1.7 ± 2.1	0.030
Symptoms									
Symptom (0-130)	9	42.0 ± 23.1	54.2 ± 20.3	12.3 ± 17.4	10	31.7 ± 18.6	46.5 ± 23.3*	14.8 ± 19.3	0.77
Interference (0-60)	9	23.2 ± 18.3	29.8 ± 13.7	6.6 ± 14.9	10	24.6 ± 10.0	26.7 ± 19.4	2.1 ± 19.1	0.58
Recovery									
Platelet transfusion (ml)	10	987.2 ± 856.2			11	1243.8 ± 1208.8			0.59
Red blood cell transfusion (ml)	10	368.9 ± 428.1			11	807.3 ± 875.1			0.17
Platelet engraftment (day)	10	11.5 ± 6.9			11	12.9 ± 6.5			0.64

N/A, not applicable; EG, exercise group; CG, control group.

p value for comparison of change of variables between exercise and control group.

* p <0.05 vs. baseline.

### Step 8. Focus group interview

The result of this process has previously been reported (26). Through this process, three exercise barriers and four exercise facilitators were identified. The exercise barriers included physical barriers such as nausea, vomiting, sore throat, reduced appetite; psychological barriers such as decreased willpower and anxiety due to isolation; and environmental barriers such as neighboring patients’ negative opinions about the exercise program and a lack of encouragement from the medical professionals. Exercise facilitators included the desire to exercise; easy, simple, and safe nature of the exercises; and the availability of detailed and reliable information about the importance and potential benefits of exercise from the medical professionals rather than an exercise therapist. Finally, the participants preferred the exercise program to be supervised by the exercise therapists rather than unsupervised.

### Final exercise program

Despite the participants preferring a supervised program, the supervised sessions were provided only before the isolation period started due to limited access to the isolated room. Instead, a brochure including pictures of and instructions for each movement and online videos viewable by smartphone for self-managed sessions during the isolation. The exercise program is shown in **Tables 4**, **5**. The exercise program is daily and included stretching and resistance exercises using body weight in three different positions (i.e., lying down, sitting, and standing). The exercise intensity was classified as very low, low, or moderate, among which the participants were allowed to choose depending on their daily condition. The choice of the sitting or standing position was decided according to the room space. One to two sets of 10 repetitions for all isotonic exercise and 10 seconds for all isometric exercises and stretches were performed each day. If the participants had osteolytic lesions, they were prescribed extra exercises for the problematic joint. The daily maximum exercise time was less than 30 minutes, which was shorter than previous exercise programs considering the patients’ low physical condition and daily exercise frequency.

**Table 4:** Contents of inpatient exercise program for HSCT patients.

Exercise type		Detailed exercises
Stretching in a supine position (very low intensity)		Neck and ankle stretching Shoulder circle Trunk rotation Hamstring stretching Hip stretching Thigh isometric exercise
Calisthenics exercise in a supine position (low intensity)		Neck bridge Lying band front raise Lying band arm curl Posterior pelvic tilt Shoulder bridge Ball squeeze (hip adduction) Calf raise against bed frame
Calisthenics exercises mostly in sitting position (moderate intensity)		Sitting band row Kneeling wall push-up Crunch Straight-leg bridge on bed Side leg raise Sitting thigh isometric exercise Calf raise against bed frame
Calisthenics exercises mostly in standing position (moderate intensity)		Standing band row Standing bed push-up Crunch Good morning and side band exercise Standing hip exercise (abduction, flexion, and extension) Sit and stand Standing calf raise
Joint exercise	Shoulder	Lying band external-rotation exercise Lying band internal-rotation exercise
	Lower back	Posterior pelvic tilt (focus on transverse abdominis muscle) Shoulder bridge
	Knee	Lying thigh isometric exercise Sitting isometric leg curl

HSCT, hematopoietic stem cell transplantation.

**Table 5:** Exercise program tailored to the patient’s daily condition and room type.

Room	Condition		
	Bad (very low intensity)	Normal (low intensity)	Good (moderate intensity)
Laminar air flow room or General room	Stretching	Stretching and lying down resistance exercise	Stretching and standing resistance exercise
Bio-clean room	Stretching	Stretching and lying down resistance exercise	Stretching and sitting resistance exercise
Joint exercise^a^	Shoulder/lower back/knee exercise		

a Patients with joint problems add exercise for each problematic joint.

## Discussion

Although exercise is a safe intervention method, it can cause injury, tiredness, or even fainting in patients with poor health condition. Nevertheless, no reliable standardized exercise program for HSCT patients has been developed to date. This study introduced the development process of an evidence-based exercise program for HSCT patients, which is a modified version of the An et al.’s development process of the exercise program for postoperative colorectal cancer patients (21).

Through the literature review, we realized that medical environments for HSCT among different countries differ and that we needed a tailored program suitable for the Korean medical environment. In most previous studies, the participants performed aerobic exercise using a treadmill or stationary bike (5–7, 11–18) and participated in a supervised program (5–11, 14–18, 27). However, in the Korean medical environment, the use of a treadmill and stationary bike, and a supervised program were not available because of confined space and lack of access to the isolated room. For these reasons, we developed a suitable program for the Korean medical environment that included bed exercises requiring minimal exercise equipment and space. These findings suggest that the development of a tailored intervention program based on participant needs and study environments may be essential in different circumstances.

Participants in the exercise group tended to maintain their body composition, physical function, and symptoms, and to improve hematologic recovery after HSCT compared to the control group, despite the modest exercise adherence, a small sample size, and the short period of intervention. These findings are consistent with Ahn et al.’s study (28) which showed the trends of maintaining body composition and physical functions in the exercise group but decreasing in the control group only after 7 to 9 days in colorectal cancer patients after surgery. Abo et al. (29) reported a significant decline in functional exercise capacity in patients undergoing allogeneic bone marrow transplantation despite exercise intervention. Simply maintaining physical functions during HSCT is very difficult in this vulnerable population, which indicates our findings are significantly meaningful and the exercise program could be helpful for patients during HSCT. Moreover, the findings suggest that greater exercise adherence, continuous exercise after discharge, and a large sample size may be able to make significant improvements in body composition, physical function, and recovery in patients undergoing HSCT.

Low adherence to the exercise program was the most important limitation of this study. Previous studies (7, 15, 17, 30) reported 94% and 87% rates of exercise adherence with the supervised program, respectively. However, we were unable to use the supervised program because of the inaccessibility during the isolation period; thus, our adherence rate was much lower (45%). In the focus group interview, the participants mentioned poor physical and psychological conditions as exercise barriers; and the easy and simple nature of the exercises, and reliability of the information. Many participants admitted that the exercise program was easy and simple to perform. Nevertheless, they may not be motivated when their physical and psychological conditions are extremely poor due to chemotherapy and HSCT.

Participants also reported a lack of motivation from medical professionals as an exercise barrier. According to the Theory of Planned Behavior, if they think their significant others want them to perform the behavior (subjective norm), they will have the intention to perform the behavior (31). Furthermore, Park et al. (32) showed that an oncologist’s exercise recommendation with an exercise motivation package significantly increased exercise participation among breast and colorectal cancer patients. If not only exercise therapists but also medical professionals such as physicians or nurses provided exercise recommendations and encouragements, it might have been able to improve exercise adherence and show significant differences between groups.

Limitations of this study include the low exercise adherence rate, small sample size, lack of data on recruitment rate in the pilot RCT, and non-significant differences between groups in the feasibility study. Nevertheless, this study showed the detailed process of development of the exercise program for HSCT patients using evidence from the literature, expert opinions, patient feedback, and objective measures. Furthermore, this study demonstrated the potential of the developed program to improve the health-related fitness and recovery of HSCT patients. A randomized controlled trial using this evidence-based exercise program with additional support by medical professionals is required to validate its effects.

In conclusion, this study described the development process of an evidence-based exercise program for Korean HSCT patients. Despite evidence from the literature review, patient needs, and expert opinions, exercise adherence was low in the real clinical setting. To validate the effects of this evidence-based exercise program, a randomized controlled trial with a large sample size and strategies to improve exercise adherence involving medical professionals’ cooperation are required. If adherence to the program is improved, the developed evidence-based exercise program may provide benefits in maintaining physical functions and improving hematologic recovery in patients undergoing HSCT. Furthermore, the development process shown in this study would provide researchers and medical practitioners with fundamental information to develop an evidence-based program for patients with different diseases.

## Data availability statement

The raw data supporting the conclusions of this article will be made available by the authors, without undue reservation.

## Ethics statement

All study procedures were approved by the institutional review board and the research ethics committee of Severance Hospital (no. 4-2016-0676). Written informed consent was obtained from all participants.

## Author contributions

JJ, J-WC, M-SY, and K-YA participated in the study concept and design. J-WC and MC participated in participants recruitment. K-YA had full access to all of the data in the study and participated in data analysis. K-YA, M-SY, and MC participated in data collection. K-YA participated in the drafting of the manuscript. WC, MC, KC, J-WC, and JJ participated in critical revision of the manuscript. All authors contributed to the article and approved the submitted version.
