# Supplementary material for: An 8-step approach for the systematic development of an evidence-based exercise program for patients undergoing hematopoietic stem cell transplantation
Source: Front Oncol. 2023 Apr 18;13:1132776. doi: 10.3389/fonc.2023.1132776 (PMC10153648; doi:10.3389/fonc.2023.1132776)
Supplement: Supplementary file 1 [file Table_1.pdf]

Supplementary Table 1. Characteristics and exercise programs of the selected studies

Author (year)	Design	Participants	Intervention	Exercise program details	Result
Cunningham B.A. et al. (1986)	Three-arm RCT: exercise 3 times/week (EG3) vs. exercise 5 times/week (EG5) vs. control (CG)	30 marrow transplantation: EG3 = 10, EG5 = 10, CG = 10	EG: resistance exercise	Resistance exercise: biceps- triceps curl, bench press, shoulder retractors, straight leg raise, hip extension, hip abduction, sit up, knee extension;<br>30 min/session; 15 reps	Body weight: ↔<br>Arm muscle area: ↔<br>Temperature: ↔<br>Nitro balance: ↔<br>Creatinine excretion: ↓
Dimeo F. et al. (1997)	Two-arm RCT: exercise (EG) vs. control (CG)	70 patients underwent chemotherapy for auto-PBSCT: EG = 33, CG = 37	EG: daily aerobic exercise	Bed ergometer: 1 min, at least 50% of the cardiac reserve ×15 times, 1-min rest between bouts,<br>30 min/day, daily	Physical performance: CG↓ vs. EG<br>Neutropenia: EG↓ vs. CG<br>Thrombopenia: EG↓ vs. CG<br>Platelet transfusion: EG↓ vs. CG<br>Cardiologic examination: ↔<br>Pain severity: EX↓ vs. CG<br>Duration of hospitalization: EG↓ vs. CG
Mello M. et al. (2003)	Two-arm RCT: exercise (EG) vs. control (CG)	18 allo-BMT patients: EG = 9, CG = 9	EG: active exercise, muscle stretching, treadmill walking	Active ROM exercise: shoulder, elbow, hip, knee and ankle<br>Stretching: hamstring, triceps surae, quadriceps muscle.<br>Treadmill walking: 5 sets of 3 min, 3-min rest between sets in the first week, 2 sets of 10 min with accelerated in the 6th week;<br>daily, 40 min/session, HR < 70% of HR_{max}, 6 weeks	Muscle strength: EG↑ vs. CG
Kim S.D. & Kim H.S. (2005)	Two-arm RCT:	35 allo-BMT patients: EG = 18,	EG: bed exercise	Bed exercise: preliminary exercise for 10 min, relaxation breathing for	Fatigue: EG↓ vs. CG

	exercise (EG) vs. control (CG)	CG = 17		10 min, finish exercise for 10 min<br>Preliminary exercise: concentrate the attention on lower abdomen for 3 min, put left ankle on right knee for 3 min, put right ankle on left knee for 2 min, bend both knees for 2 min<br>Finish exercise: rest and relax for 2 min, stroke hair and face for 2 min, rotate both ankles right and left for 2 min, stretch legs and arms for 2 min, stretch out on the bed for 2 min<br>Every day, 30 min, 6 weeks	
Kim S.D. & Kim H.S. (2005)	Two-arm RCT: exercise (EG) vs. control (CG)	35 allo-BMT patients: EG = 18, CG = 17	EG: bed exercise	Bed exercise: preliminary exercise for 10 min, relaxation breathing for 10 min, finish exercise for 10 min<br>Preliminary exercise: concentrate on lower abdomen for 3 min, put left ankle on right knee for 3 min, put right ankle on left knee for 2 min, bend both knees for 2 min<br>Finish exercise: rest and relax for 2 min, stroke hair and face for 2 min, rotate both ankles right and left for 2 min, stretch legs and arms for 2 min, stretch out on the bed for 2 min<br>Every day, 30 min, 6 weeks	Anxiety: EG↓ vs. CG<br>Depression: EG↓ vs. CG<br>Leukocyte: EG↑
Kim S.D. & Kim H.S. (2006)	Two-arm RCT: exercise (EG) vs. control (CG)	35 allo-BMT patients: EG = 18, CG = 17	EG: bed exercise	Bed exercise: preliminary exercise for 10 min, relaxation breathing for 10 min, finish exercise for 10 min<br>Preliminary exercise: concentrate on lower abdomen for 3 min, put	Lymphocyte: CG↓ vs. EG<br>T-cell subset: ↔

				left ankle on right knee for 3 min, put right ankle on left knee for 2 min, bend both knees for 2 min<br>Finish exercise: rest and relax for 2 min, stroke hair and face for 2 min, rotate both ankles right and left for 2 min, stretch legs and arms for 2 min, stretch out on the bed for 2 min<br>Every day, 30 min, 6 weeks	
DeFor T.E. et al. (2007)	Two-arm RCT: exercise (EG) vs. control (CG)	100 allo-HCT patients: EG = 51, CG = 49	EG: structured walking regimen<br>CG: no formal exercise	Treadmill walking: 2 times/day, at least 15 min during hospitalization; 1 time/day, 30 min after discharge	PA level: EG↑ vs. CG<br>Karnofsky: ↔ in total population, CG↓ vs. EG in patients who received nonmyeloablative conditioning<br>Physical and emotional well-being: EG↑ vs. CG , ↔ at 100 days post-transplant<br>Length of hospital stay: ↔<br>Survival: ↔
Jarden M. (2007)	Two-arm RCT: exercise (EG) vs. control (CG)	19 allo-HSCT patients (14 completed): EG = 8, CG = 6	EG: multimodal intervention of exercise, progressive relaxation, and psychoeducation<br>CG: conventional care	Stationary cycling: 5 days/week, low to moderate intensity (50-75% HR_{max}, RPE 10-13, 5.5 MET), 15-30 min/session, rest intervals as needed, increase intensity and duration<br>Stretching: 5 days/week (dynamic: 1-2 sets, 10-12 reps, static: 1 set, hold for 15-30 sec), 3.5 MET, 15-20 min/session<br>Resistance training: 3 days/week, low to moderate intensity (1-2 sets, 10-12 reps, RPE 10-13, 3 MET), 15-20 min/session	Feasibility: 74% completed<br>Exerciser group: 66% completed<br>Adherence: 94% (cycling 86%, dynamic stretching 88%, resistance and relaxation 100%)<br>Chest press: EG↑ vs. CG<br>Leg extension: EG↑ vs. CG<br>Right knee flex: EG↑ vs. CG<br>Right elbow: ↔<br>$VO_{2max:}$ ↔<br>Stair test: ↔

				Progressive relaxation: 2 days/week, low intensity (RPE: 6-9. 2.5 MET), 20 min/session	
Jarden M. et al. (2009)	Two-arm RCT: exercise (EG) vs. control (CG)	42 allo-HSCT patients: EG = 21, CG = 21	EG: multimodal intervention of exercise, progressive relaxation, and psychoeducation<br>CG: conventional care	Stationary cycling: 5 days/week, low to moderate intensity (50-75% HR_{max}, RPE 10-13, 5.5 MET), 15-30 min/session, rest intervals as needed, increase intensity and duration<br>Stretching: 5 days/week (dynamic: 1-2 sets, 10-12 reps; static: 1 set, hold for 15-30 sec), 3.5 MET, 15-20 min/session<br>Resistance training: 3 days/week, low to moderate intensity (1-2 sets, 10-12 reps, RPE 10-13, 3 MET), 15-20 min/session<br>Progressive relaxation: 2 days/week, low intensity (RPE: 6-9, 2.5 MET), 20 min/session	VO_{2max}: EG↑<br>Muscle strength: EG↑<br>Diarrhea (EORTC): EG↓<br>FACT-An: ↔<br>HADS: ↔<br>Self-reported PA: ↔
Jarden M. et al. (2009)	Two-arm RCT: exercise (EG) vs. control (CG)	42 allo-HSCT patients: EG = 21, CG = 21	EG: multimodal intervention of exercise, progressive relaxation, and psychoeducation<br>CG: conventional care	Stationary cycling: 5 days/week, low to moderate intensity (50-75% HR_{max}, RPE 10-13, 5.5 MET), 15-30 min/session, rest intervals as needed, increase intensity and duration<br>Stretching: 5 days/week (dynamic: 1-2 sets, 10-12 reps; static: 1 set, hold for 15-30 sec), 3.5 MET, 15-20min/session<br>Resistance training: 3 days/week, low to moderate intensity (1-2 sets, 10-12 reps, RPE 10-13, 3 MET), 15-20 min/session	Symptom: EG↓ vs. CG

				Progressive relaxation: 2 days/week, low intensity (RPE: 6-9. 2.5 MET), 20 min/session	
Baumann F.T., (2009)	Two-arm RCT: exercise (EG) vs. control (CG)	64 HSCT patients: EG = 32, CG = 32	EG: endurance + activities of daily living (ADL) training<br>CG: standard care	Endurance: cycle ergometer, 7 times/week, goal: 10-20 min without interruption, watt load increased by 25 watt every 2 min until the patient reached a set heart rate (180 - age). Achieved watt load decreased by 20% and defined as patient's training intensity.<br>ADL: strength, coordination, stretching, walking, ad stair climbing, 20-30 min/day, "slightly strenuous" or "strenuous" on the Borg scale.	Endurance: CG↓ vs. EG<br>Strength: CG↓ vs. EG<br>QOL (EORTC): ↔<br>Hematology: ↔
Wiskemann J. et al. (2010)	Two-arm RCT: exercise (EG) vs. control (CG)	105 allo-HCT patients (80 completed): EG = 52, CG = 53	EG: home-based exercise (1-4 weeks) before admission, supervised exercise after admission, home-based exercise after (8 weeks) discharge<br>Endurance and resistance exercise using stretch bands<br>CG: moderate PA recommendation, Telephone calls and visits in	Endurance: 3-5 times/week, 20-40 min/session<br>Resistance: 2 times/week 20-40 min/session	Fatigue (MFI, POMS): CG↑ vs. EG<br>Fatigue (EORTC): ↔<br>Physical function (EORTC, HADS): EG↑ vs. CG<br>Anxiety (HADS): EG↑<br>Distress (HADS): EG↓<br>Anger/hostility (POMS): EG↑ vs. CG<br>Pain (EORTC): EG↓ vs. CG<br>6-MWT: EG↑<br>Lower-extremity strength: EG↑<br>Pedometer step: ↔<br>Coordination (balance): ↔

			hospital as in EG		
Baumann F.T. et al. (2011)	Two-arm RCT: exercise (EG) vs. control (CG)	47 allo-HSCT(33 completed, 14 deceased): EG = 17, CG = 16	EG: endurance + ADL training<br>CG: standard care	Endurance: cycle ergometer, 7 times/week, goal: 10-20 min without interruption, watt load increased by 25 watt every 2 min until the patient reached a set heart rate (180 - age). Achieved watt load decreased by 20% and defined as patient's training intensity.<br>ADL: strength, coordination, stretching, walking, and stair climbing, 20-30 min/day, "slightly strenuous" or "strenuous" on the Borg scale.	Endurance: CG↓<br>Strength: CG↓<br>Lung function: ↔<br>Weight: EG↓CG↓<br>Fatigue: CG↑<br>Physical function: EG↓CG↓<br>Emotional state: EG↑
Oechsle K. et al. (2014)	Two-arm RCT: exercise (EG) vs. control (CG)	48 auto-PBSCT: EG = 24, CG = 24	EG: supervised exercise, warm-up + endurance + resistance exercise + stretching	Endurance: bicycle ergometer, 5 times/week, 10-12 min/session, individually adjusted intensity<br>Resistance: bridging, sit-ups, back and arm exercise using elastic band, 40-60% of 1RM, 16-25 reps, 2 sets, 5 times/week, 20 min/session	Physical performance: EG↑ CG↓<br>Oxygen consumption: EG↑ vs. CG<br>Expiratory minute ventilation: EG↑ vs. CG<br>QOL (physical function): EG↑ vs. CG<br>Fatigue (cognition, psychosocial function): EG↓ vs. CG

Wiskemann J. et al. (2013)	Two-arm RCT: exercise (EG) vs. control (CG)	105 allo-HCT patients (80 completed): EG = 52, CG = 53	EG: home-based exercise (1-4 weeks) before admission, supervised exercise after admission, home-based exercise (8 weeks) after discharge<br>Endurance and resistance exercise using stretch bands<br>CG: moderate PA recommendation, Telephone calls and visits in hospital as in EG	Endurance: 3-5 times/week, 20-40 min/session<br>Resistance: 2 times/week 20-40 min/session	6MWT: EX unfit ↑ vs. EX fit<br>All muscle strength group: EX unfit ↑ vs. EX fit<br>Knee-extension: Unfit ↑ vs. control<br>Hip-flexion: Unfit ↑ vs. control<br>Elbow-extension: Unfit ↑ vs. control<br>6MWT: Unfit ↑ vs. control
Jacobsen P.B. et al. (2014)	Four-arm RCT: exercise (EG) vs. stress management (SM) vs. combination(COM) vs. usual care (CG)	711 auto- or allo-HCT patients: EG = 180, SM = 178, COM = 178, CG = 175	EG: walking<br>SM: abdominal breathing + muscle relaxing + coping self-statements<br>COM: exercise + stress management	Exercise goal: 20-30 min/session, 3-5 times/week, 50-75% of heart rate reserve, 180 days	PSC (+100 days): ↔<br>MCS (+100 days): ↔<br>Overall survival (+100 days): ↔<br>Days of hospitalization (+100 days): ↔<br>Distress (+100 days): ↔<br>Sleep quality (+100 days): ↔<br>Pain (+100 days): ↔<br>Nausea (+100 days): ↔
Bargi G. et al. (2016)	Two-arm RCT: inspiratory muscle training (IMT) group	55 allo-HSCT patients (38 completed): IMT = 20, CG = 18	IMT: IMT at 40% of maximal inspiratory pressure (MIP)<br>CG: IMT at 5% of MIP	30 min/day, 7 days/week, 40% of MIP, 6 weeks	MISWT: IMT↑ vs. CG<br>6-MWT: IMT↑ vs. CG<br>Respiratory muscle strength: IMT↑ vs. CG<br>Depression: IMT↓ vs. CG<br>Dyspnea: IMT↓

	vs. control group (CG)				Fatigue: IMT↓<br>Peripheral muscle strength: ↔<br>QOL: IMT↑
Kuehl R. et al. (2016)	Two-arm RCT: exercise (EG) vs. control (CG)	153 allo-HCT patients: EG = 76, CG = 77	EG: endurance and resistance exercise<br>CG: muscle relaxation program	5 times/week (3 supervised) during hospitalization<br>Home-based 3-5 times/week after discharge (10-min phone call)	Adherence: 100%<br>Determinants for exercise adherence: low physical fatigue, having children home during inpatient, endurance performance, controlling for emotional functioning after discharge<br>Determinants for contamination: isometric muscle strength, physical fatigue

6MWT: 6-min walking test; allo-HCT, allogeneic hematopoietic cell transplantation; BMT, bone marrow transplantation; HSCT, hematopoietic stem cell transplantation; MISWT: Modified Incremental Shuttle Walking Test; PBSCT, peripheral blood stem cell transplantation; RM, repetition maximum; ROM, range of motion
